# Contrast-enhanced micro-CT imaging in murine carotid arteries: a new protocol for computing wall shear stress

**DOI:** 10.1186/s12938-016-0270-2

**Published:** 2016-12-28

**Authors:** Ruoyu Xing, David De Wilde, Gayle McCann, Yanto Ridwan, Jelle T. C. Schrauwen, Anton F. W. van der Steen, Frank J. H. Gijsen, Kim Van der Heiden

**Affiliations:** 1 Department of Biomedical Engineering, Thorax Center, Erasmus MC, Wytemaweg 80, Ee2338, 3015CN, Rotterdam, The Netherlands; 2 IBiTech-bioMMeda, iMinds Medical IT, Ghent University, De Pintelaan 185, 9000 Ghent, Belgium; 3 Department of Genetics, Erasmus MC, Wytemaweg 80, Ee720, 3015CN, Rotterdam, The Netherlands

**Keywords:** Atherosclerosis, Wall shear stress, Micro-CT imaging, Contrast media, Image segmentation

## Abstract

**Background:**

Wall shear stress (WSS) is involved in the pathophysiology of atherosclerosis. The correlation between WSS and atherosclerosis can be investigated over time using a WSS-manipulated atherosclerotic mouse model. To determine WSS in vivo, detailed 3D geometry of the vessel network is required. However, a protocol to reconstruct 3D murine vasculature using this animal model is lacking. In this project, we evaluated the adequacy of eXIA 160, a small animal contrast agent, for assessing murine vascular network on micro-CT. Also, a protocol was established for vessel geometry segmentation and WSS analysis.

**Methods:**

A tapering cast was placed around the right common carotid artery (RCCA) of ApoE^−/−^ mice (n = 8). Contrast-enhanced micro-CT was performed using eXIA 160. An innovative local threshold-based segmentation procedure was implemented to reconstruct 3D geometry of the RCCA. The reconstructed RCCA was compared to the vessel geometry using a global threshold-based segmentation method. Computational fluid dynamics was applied to compute the velocity field and WSS distribution along the RCCA.

**Results:**

eXIA 160-enhanced micro-CT allowed clear visualization and assessment of the RCCA in all eight animals. No adverse biological effects were observed from the use of eXIA 160. Segmentation using local threshold values generated more accurate RCCA geometry than the global threshold-based approach. Mouse-specific velocity data and the RCCA geometry generated 3D WSS maps with high resolution, enabling quantitative analysis of WSS. In all animals, we observed low WSS upstream of the cast. Downstream of the cast, asymmetric WSS patterns were revealed with variation in size and location between animals.

**Conclusions:**

eXIA 160 provided good contrast to reconstruct 3D vessel geometry and determine WSS patterns in the RCCA of the atherosclerotic mouse model. We established a novel local threshold-based segmentation protocol for RCCA reconstruction and WSS computation. The observed differences between animals indicate the necessity to use mouse-specific data for WSS analysis. For our future work, our protocol makes it possible to study in vivo WSS longitudinally over a growing plaque.

## Background

Atherosclerosis is an inflammatory disease characterized by the accumulation of lipids, fibrous tissue and inflammatory cells [1, 2]. Atherosclerotic plaques are predisposed to develop at the inner curvatures and branches of the arterial system, a process which is known to be driven by the local wall shear stress (WSS) environment [3, 4]. WSS is the frictional force that blood flow exerts on the endothelium of the vessel wall. The endothelial cells respond to various WSS patterns differently by inducing either an anti-inflammatory or a pro-inflammatory profile [5]. The pro-inflammatory status of the endothelial cells thus primes the vessel wall for atherosclerotic development [3]. As the disease progresses, the plaque intrudes into the lumen, thereby altering the local WSS distribution. It has been hypothesized that WSS is also involved in plaque progression [4, 6, 7] yet there is no in vivo animal experimental evidence. In order to study the effect of different WSS patterns on plaque progression in vivo, a WSS-induced atherosclerotic mouse model has been developed [8]. In this model, a tapering cast is placed around the animal’s right common carotid artery (RCCA), inducing distinct WSS patterns in this vessel. As a result, atherosclerotic plaques developed both upstream and downstream to the cast [8, 9].

The most commonly used method to determine WSS on the vessel wall is computational fluid dynamics (CFD). CFD is a well-established computational tool that computes the velocity and pressure of the blood in a vessel. WSS is subsequently derived from the velocity field. Previous studies have used CFD to illustrate the WSS patterns in the murine vasculature [10–14]. In order to accurately calculate WSS, reliable three-dimensional (3D) geometry of the blood vessel is required. The use of contrast agents in micro-CT imaging allows blood vessels as small as 100 µm to be visualized [15, 16], which makes it possible to obtain an accurate 3D vessel geometry for CFD-based WSS analysis in mice.

Several commercially available contrast agents for use in small animals have been tested for evaluating vascular structures in healthy and atherosclerotic mice. Monitoring of atherosclerosis progression ideally requires longitudinal studies. Such studies require multiple micro-CT imaging and contrast injections over time. Therefore, it is necessary to use a contrast agent that provides good signal enhancement, at the same time, is biocompatible with the murine system. We compared different small animal contrast agents based on their signal enhancement and potential adverse effects.

Fenestra VC (MediLumine) was the first iodine-based contrast agent developed for murine vascular imaging. It has a low iodine concentration (50 mg/ml) and thus low blood-pool enhancement [17]. AuroVist 15 nm (nanoprobes) is a gold nanoparticle that gives excellent signal enhancement. However, this contrast agent leaves dark staining at the injection spot, making it extremely difficult to distinguish the vein for repeated administration [15]. Also, histology analysis has showed that AuroVist 15 nm is taken up by plaques [18], which might interfere with repeated measurements. These properties limit the use of AuroVist 15 nm for studying atherosclerosis progression regardless of the strong signal it provides. Exitron nano 12,000 (Miltenyi Biotec) is a barium-based nanoparticle with polymer coating [16]. Its high equivalent iodine concentration (300 mg/ml) permits excellent contrast [19]. However, Exitron nano 12,000 is cleared via macrophages [19, 20] and accumulates in liver and spleen and is still present after months, allowing possible imaging even 185 days after a single contrast injection [21]. This would potentially complicate longitudinal vessel imaging. Since macrophages are involved in the pathogenesis of atherosclerosis [2], Exitron nano 12,000 might accumulate in plaques and give false signals during subsequent lumen measurements, leading to an overestimation of lumen surface and subsequently an underestimation of WSS. Moreover, repeated injections have been shown to have toxic effects [22]. This leaves our choice to the recently developed eXIA 160 and eXIA 160XL (Binitio Biomedical), which are aqueous colloidal polydispersed contrast materials. Both contrast agents have a high iodine concentration (160 mg/ml), allowing for good signal enhancement. eXIA 160XL remains in the vascular circulation longer than eXIA 160 does [23, 24]. Because current imaging protocols allow fast image acquisition [25], prolonged vascular circulation of eXIA 160XL is not necessary. A second property of eXIA 160XL that makes it less suitable for longitudinal studies than eXIA 160 is that data on survival rates after the use of eXIA 160XL are inconclusive, since separate studies have reported 50% [15] or 100% [24] survival rates. On the contrary, several studies have suggested that biocompatibility of eXIA 160 with the murine system is excellent, as the contrast agent was eliminated from the blood, liver and spleen within 24 h [17, 23]. Given the high contrast intensity of eXIA 160 and its biocompatibility, eXIA 160 is the only contrast agent promising for the purpose of longitudinal studies.

The aim of this study was to determine whether eXIA 160 is a suitable contrast agent for reconstructing reliable 3D geometry for CFD analyses in the WSS-manipulated atherosclerotic mouse model. As the vessel narrows and become severely stenotic due to atherosclerotic plaque progression, it is challenging to capture vessel geometry using this specific animal model. Contrast-enhanced micro-CT was performed on atherosclerotic mice using eXIA 160 and contrast enhancement in the RCCA and the surrounding soft tissue by eXIA 160 was subsequently analyzed. At the same time, we investigated a semi-automatic segmentation procedure to reconstruct 3D murine vessel geometry. We established a standardized protocol for local threshold-based segmentation and we compared this protocol with a global threshold segmentation method. Finally, transient CFD analyses using mice-specific flow data were performed to illustrate how the segmentation methods influence the computed WSS distribution.

## Methods

### Animal preparation

Female ApoE^−/−^ mice on C57BL/6 J background (n = 8) were purchased from Charles River (Maastricht, The Netherlands) and were fed normal chow diet up to 13 weeks of age. An atherogenic Western diet (nr 4021.06, Arie Blok, Woerden, The Netherlands) was then introduced and provided ad libitum. At 15 weeks of age, cast surgery was performed on the animals under isoflurane-induced anesthesia. A tapering cast was placed around the right common carotid artery (RCCA) of the animal as described previously [8, 26]. The cast has a tapering lumen (diameter from 400 to 200 µm over 1.5 mm) which changed the blood flow along the RCCA, intended to induce a decreased shear stress region upstream of the cast and an oscillatory shear stress area downstream of the cast. All the animal experiments were approved and conducted according to guidelines by the ethical committee of Erasmus MC Rotterdam.

### Doppler ultrasound

Five weeks after cast placement, mice were prepared for Doppler ultrasound imaging. Blood flow through the RCCA was imaged with a high-frequency ultrasound scanner (Vevo 2100, VisualSonics Inc., Toronto, Ontario, Canada) using a 40-MHz transducer. Doppler velocity measurements were recorded in the RCCA upstream of the cast. Five consecutive cycles were recorded, and averaged to obtain the transient flow wave form for the CFD simulations.

### Contrast agents and micro-CT imaging

Directly after Doppler ultrasound, mice were prepared for micro-CT imaging. Both imaging were performed during the same anesthesia session. A commercially available contrast agent developed for murine studies, namely eXIA 160 (Binitio Biomedical) was tested. We injected as much contrast as allowed in our animals because we intended for the highest possible amount of contrast circulating in the murine system for image segmentation. The maximum injection volume was 150 µl according to the local animal welfare regulations. In order to correct the contrast concentration in each animal, we used the maximum weight observed from the 8 animals, which was 25 g. This leaded to an injection dose of 150 µl/25 g of body weight. The contrast agent was slowly injected intravenously via the lateral caudal vein. Micro-CT imaging was performed using an ultra-fast micro-CT scanner (Quantum FX, PerkinElmer). The micro-CT acquisition parameters were 90 kvp, 160 µA with field of view 20 mm and 360° rotation in 1 step. The acquisition time was 4.5 min. Images in Hounsfield unit (HU) were reconstructed in cubic voxels of 40 µm, generating a matrix of 512 × 512 × 512 voxels. The bones and vascular network were visualized using the volume renderer available from MeVisLab (MeVisLab 2.2.1, MeVis Medical Solutions AG).

### Image segmentation

Image segmentation of the RCCA was performed on two software platforms: MeVisLab 2.2.1 and Matlab (Matlab 2011b, MathWorks, Inc.). The region of interest spanned from the separation of the RCCA from the brachiocephalic artery to the bifurcation of the internal and external carotid artery. Initial segmentation was performed in MeVisLab using two in-house developed modules [27, 28]. First, center points of the RCCA were manually identified to create the centerline of the RCCA. 2D planes that cut through the long axis of the RCCA were defined by two adjacent points on the centerline. The centerline was then straightened and the 2D planes were projected. The border of the RCCA along the long axis was then placed manually at three equally spaced rotations of the projected plane: 0°, 60° and 120°. The information of the vessel border was reconstructed by the module using spline interpolation to create an initial set of contours for RCCA geometry and subsequently exported into Matlab 2011b to analyze contrast related parameters.

**Fig. 1:**
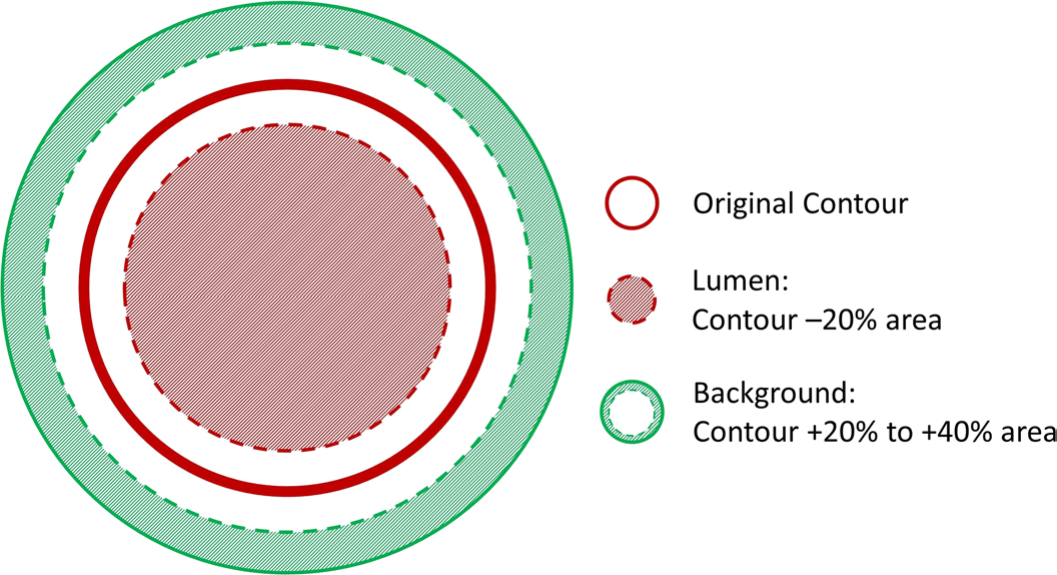
Illustration of image segmentation. *Red straight line* indicates the original contour placed in MeVisLab; Lumen area is represented by the *red shaded area* by shrinking 20% of the original contour area; Background area is defined as the area between the 20 and 40% expanded original contour, shown as the *green shaded area*

The lumen region was defined by shrinking the initial contours with 20% of their radius. Similarly, the initial contour radius was expanded with 20–40%, creating an area outside the lumen delineated by the two layers. This second region represents the surrounding background. For areas outside the cast region, soft tissue (muscle, fat tissue, etc.) was present in the background region. For areas within the cast, the cast region itself was defined as the background (Fig. 1). We determined the 20% shrinkage and 20–40% expansion based on the smallest diameter in the cast region. The diameter of the distal cast is 200 µm. The resolution of the micro-CT is 40 µm. Therefore, there are 5 pixels at the narrowest part of the vessel. While 20% of 5 pixels equals to 1 pixel, by expanding/shrinking 20% of the original contour, we included/excluded at least 1 pixel in the background/lumen regions. The voxels within the lumen region and the background region were then identified. The average HU values of these voxels inside lumen and background regions were calculated. To determine the final contours, threshold-based segmentation was performed. A straightforward method was used to determine the threshold, which was defined as the midway value between the HU values of the lumen region and the surrounding background [29]. The threshold was calculated for each contour along the entire RCCA, generating an array of local threshold values specific to each contour.

Guided by the local threshold information generated with MATLAB, the CT images were converted into a set of white/black binary images. The initial contours were manually modified based on the binary images in MeVisLab. This generated the final set of contours which were used to create the RCCA lumen surface for further analysis. Vessel diameter along the RCCA was calculated. Vessel wall thickness within the cast region was defined as half of the difference between RCCA vessel diameter and cast dimension. In addition, a global threshold value was also calculated by averaging the local threshold values along the RCCA. A global threshold guided RCCA lumen surface was created, as commonly used in vascular segmentation [30, 31]. The complete image segmentation process time was approximately 60 min per vessel.

### Computational fluid dynamics

The RCCA lumen surface was further processed using the vascular modelling tool kit (VMTK 1.2, OROBIX). A volume preserving smoothing algorithm was applied. Same smoothing parameters were used for geometry reconstructed from local and global threshold method. Flow extensions 5 times the radius of the inlet or outlet were added at both ends of the vessel. Flow extensions ensure that the flow entering and leaving the vessel is fully developed, facilitating adequate flow development for later simulation.

The surface was then imported into ICEM (ICEM-CFD 14.5, Ansys, Inc.) to generate a volume mesh with tetrahedral cells. Prism layers with quadrilateral and hexahedral cells were created at the wall. Element size was locally determined based on vessel diameter and curvature, giving rise to smaller elements in narrowed vessels or higher curvature. Parameters including maximum element size and number of prism layers were optimized to obtain a mesh-independent solution. The final mesh contained 0.6 million elements for the geometry based on the local threshold method and 1.9 million elements for the geometry based on the global threshold method.

The Navier–Stokes equations were solved by computational fluid dynamics (CFD) using Fluent (Fluent 14.5, Ansys, Inc.). Blood was modeled to be incompressible and the vessel wall rigid. As inlet boundary condition, a time-dependent velocity profile was imposed, which was derived from Doppler velocity measured upstream of the cast. No-slip boundary conditions were applied at the wall, and zero pressure was used as the outlet boundary condition. Blood was modeled as a Newtonian fluid with a viscosity of 3.5 × 10^−3^ kg/m/s and a density of 1060 kg/m^3^. The Reynolds number was calculated as Re = 7. The Newtonian assumption is justified since the characteristic shear rates in the carotid artery of mice are high [32] and transient flow simulations were performed using standard numerical techniques. The results were analyzed using CFD-Post (CFD-Post 14.5, Ansys, Inc.) and Matlab 2011b. The time-averaged WSS (TAWSS) and the oscillatory shear index (OSI) [33] were computed.

### Statistics

Data are presented as mean ± SD and analyzed in Matlab. Differences between the two segmentation methods were evaluated using a two-sample *t* test.

## Results

### Contrast enhancement

**Fig. 2:**
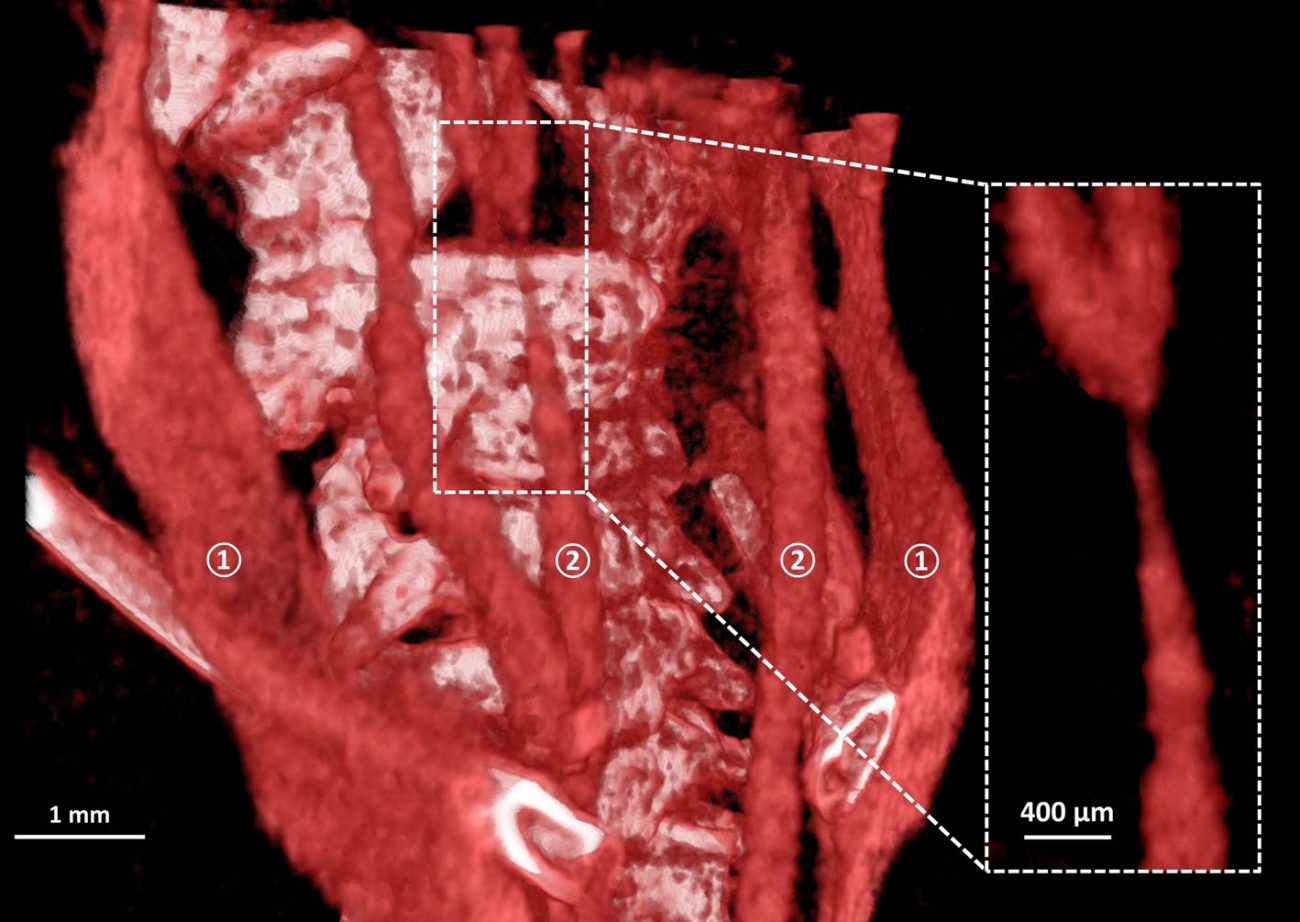
3D volume rendering of the bone structures and vascular network in the neck region for eXIA 160. Major vasculature including internal jugular vein (*①*) and the carotid arteries (*②*) were visualized (*scale bar* 1 mm). The *box* shows the zoomed-in area of the narrowing RCCA caused by the tapering cast (*scale bar* 400 µm)

A 3D volume rendering of the contrast enhancement in the lumen is shown in Fig. 2. eXIA 160 allowed visualization of the major vascular structure. The internal jugular veins (Fig. 2, Label 1) were clearly visible as they travel parallel along the vertebrae, at both sides of the neck. The common carotid arteries are located closely to the vertebrae (Fig. 2, Label 2). The narrowing of the RCCA caused by the tapering cast is clearly visible (Fig. 2, zoomed-in box).

### Contrast intensity along the RCCA

**Fig. 3:**
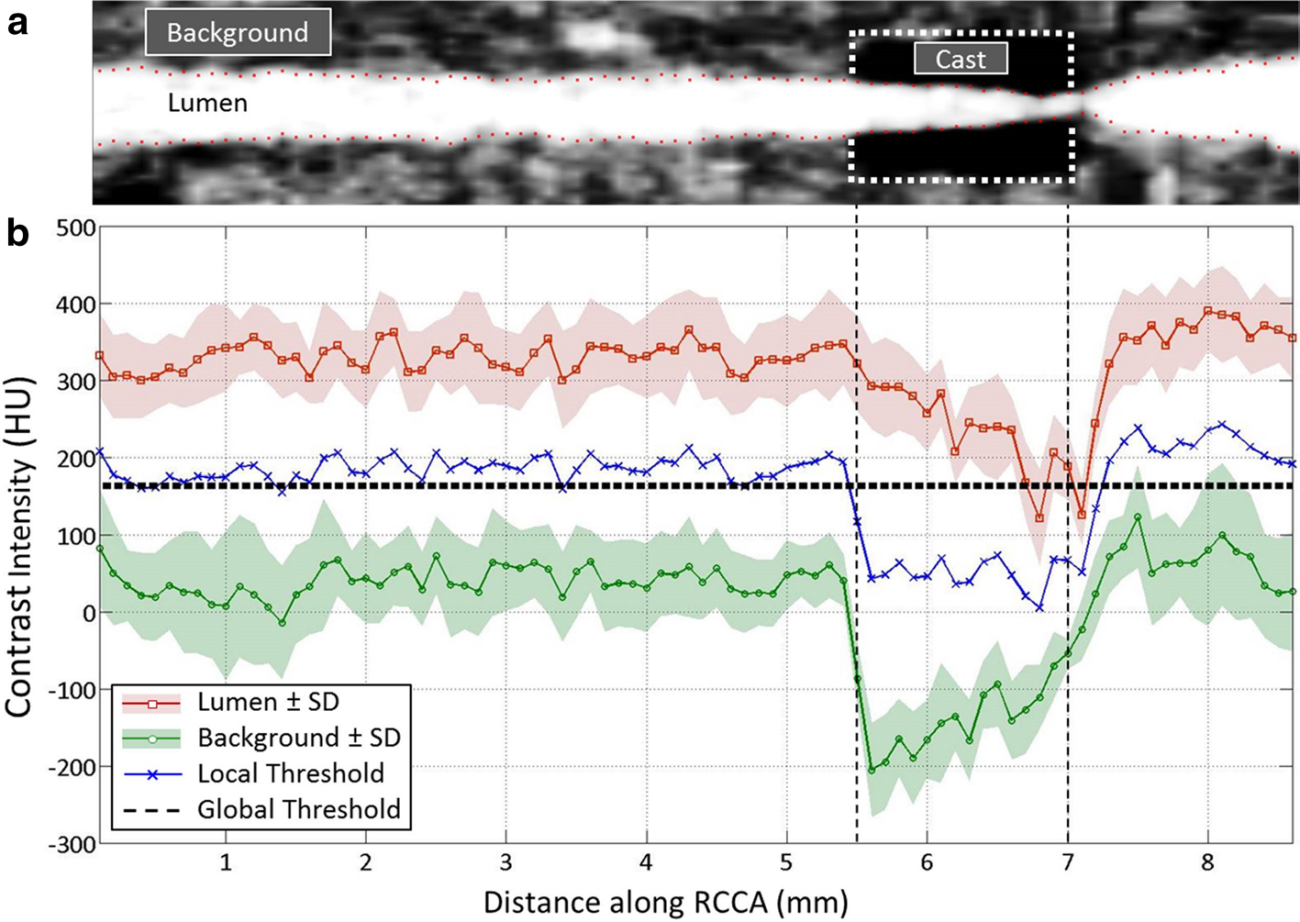
**a** Illustration of the RCCA with cast on micro-CT using eXIA 160. **b** Contrast intensity along the RCCA using eXIA 160. Lumen region is shown in *red line* with square markers; *Blue line* with cross markers represents the local threshold; *Black dashed line* indicates global threshold value; Background region is marked as green line with circles; Standard deviation is indicated by *shadow areas*

Figure 3 shows the contrast intensity of the lumen region and the background region from one of the eight mice. eXIA 160 provided good contrast intensity with a clear signal enhancement in the blood (Fig. 3b, red line with square marker) to differentiate from the surrounding background region (Fig. 3b, green line with circle marker). For this specific animal, contrast intensity was 330 HU in the lumen region and 41 HU in the background region. The difference between the two regions was 289 HU.

The contrast-related parameters of all the animals are summarized in Table 1. The average contrast intensity was 339 HU in the lumen region and 53 HU in the background region for all the eight animals. We did not observe significant difference of the contrast intensity between the lumen region upstream and downstream of the cast. The average difference between the lumen region and the background region was 285 HU, enabling visualization of the vessel lumen.

**Table 1:** Contrast-related parameters (in Hounsfield Units) of eXIA 160 along RCCA

Mouse number	Non-cast region			Cast region		
	Lumen	Background	Contrast difference	Lumen	Background	Contrast difference
1	428 ± 58	95 ± 36	323 ± 61	325 ± 90	−47 ± 51	372 ± 124
2	372 ± 35	76 ± 42	296 ± 47	299 ± 47	−93 ± 47	392 ± 87
3	286 ± 41	32 ± 54	254 ± 45	199 ± 54	−169 ± 33	368 ± 65
4	328 ± 49	67 ± 40	261 ± 41	247 ± 62	−93 ± 33	340 ± 71
5	330 ± 38	41 ± 31	289 ± 34	241 ± 53	−135 ± 45	376 ± 85
6	281 ± 33	41 ± 29	240 ± 39	191 ± 71	−96 ± 56	287 ± 111
7	306 ± 33	42 ± 35	264 ± 45	255 ± 67	−46 ± 39	301 ± 98
8	383 ± 65	31 ± 36	352 ± 67	299 ± 90	−100 ± 49	399 ± 128
Average	339 ± 51	53 ± 23	285 ± 38	257 ± 48	−97 ± 41	354 ± 41

In all the animals, the contrast intensity of the lumen region dropped within the cast region (339 ± 51 vs. 257 ± 48 HU). Similarly, a decrease in the contrast intensity of the background region was observed. The average contrast difference between the lumen region and the background region increased slightly (285 ± 38 vs. 354 ± 41 HU). Contrast-related parameters are summarized in Table 1.

Local threshold values were calculated (Fig. 3b, blue line with cross marker) by averaging the contrast intensity between the lumen region and the background region. Signal fluctuation was observed along the entire RCCA in both lumen and background region. The fluctuation was also seen along the local threshold values in all animals.

### Threshold-based segmentation

**Fig. 4:**
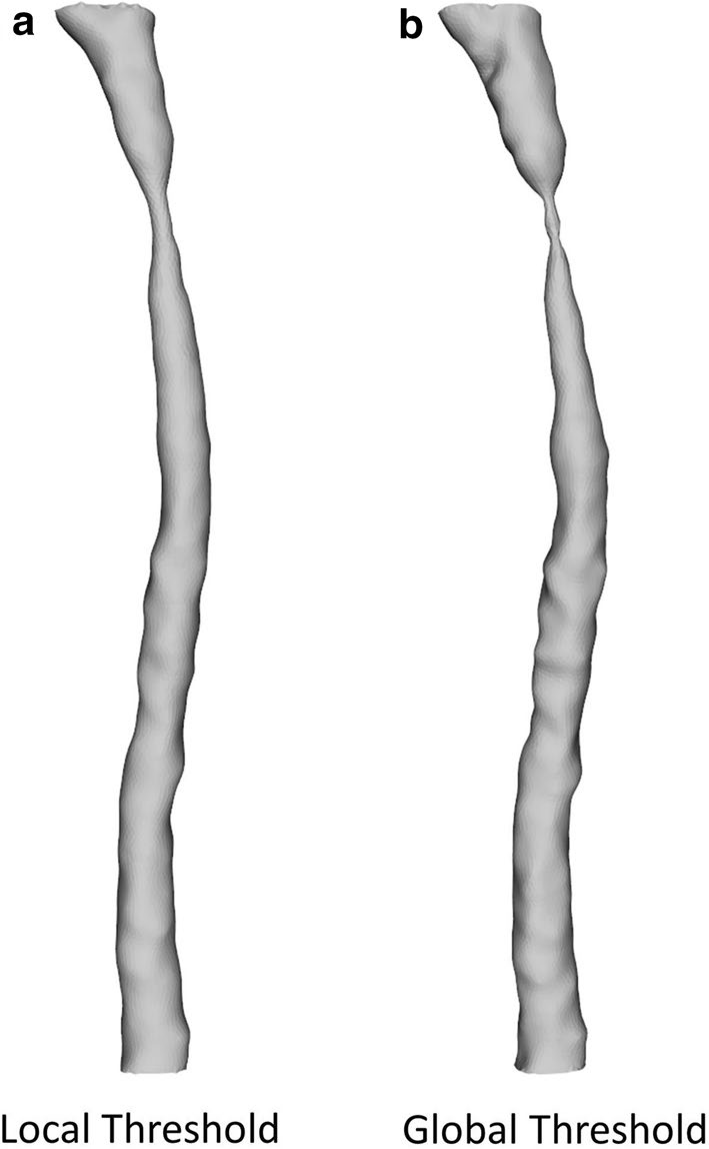
3D reconstruction of RCCA by eXIA 160 enhanced micro-CT using **a** the local threshold and **b** the global threshold segmentation. The narrowing of the lumen caused by the cast was clearly captured

**Fig. 5:**
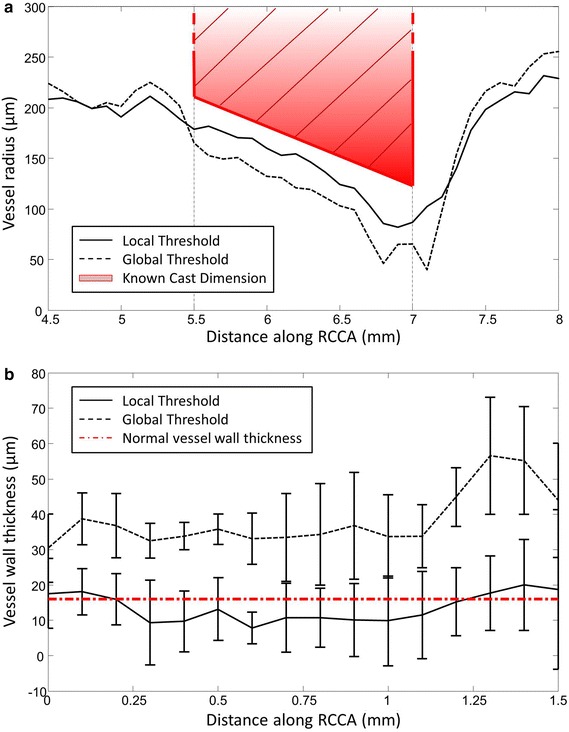
**a** Comparison of RCCA vessel dimensions obtained from two segmentation methods with known cast geometry. Vessel surface reconstructed from the local threshold segmentation (*straight line*) showed good agreement between the vessel geometry and the cast dimension. Vessel surface reconstructed from the global threshold segmentation (*dashed line*) significantly underestimated the cast dimensions. **b** RCCA vessel wall thickness within the cast region obtained from the local threshold segmentation (*straight line*) corresponded to that of the normal vessel wall thickness. Vessel surface reconstructed from the global threshold segmentation (*dashed line*) significantly overestimated the vessel wall thickness

Two sets of RCCA geometry were reconstructed using local threshold segmentation (Fig. 4a) and global threshold segmentation (Fig. 4b). The local threshold method generated smoother vessel surface than the geometry reconstructed using global threshold values. The effect of the two threshold methods on vessel geometry within the cast region was evaluated. The cast is clearly visible as a dark region surrounding the RCCA on the micro-CT image (Fig. 3a). Its diameter ranges from 400 to 200 µm, serving as a landmark for comparison. The result revealed good agreement between the cast dimension and the vessel geometry obtained from the local threshold segmentation (Fig. 5a, straight line). On average, the vessel dimension in the lumen region was 9.6 ± 4.5% smaller than that of the cast. However, the vessel geometry reconstructed from the global threshold segmentation underestimated 27.6 ± 11.6% of the lumen dimension (Fig. 5a, dashed line). As a result, the vessel was greatly narrowed in the distal cast region when using the global threshold segmentation. Vessel wall thickness reconstructed from local threshold segmentation was 13.5 ± 4.0 µm (Fig. 5b, black straight line), while global threshold methods resulted in a much larger dimension of 38.4 ± 7.9 µm (Fig. 5b, black dash line). The difference between the two methods was significant (p < 0.05). For the local threshold method, we observed thinner vessel wall thickness compared to that of healthy tissue (16.6 ± 1.3 µm, Lacolley et al. [34]). It is likely due to the reduced cyclic loading due to the placement of cast around RCCA.

For the same vessel, we performed segmentation procedures for two times. Vessel diameter of the two reconstructed geometry were calculated and compared (data not shown). There were less than 5% differences between the two segmented vessels, indicating that the vessel reconstruction was not influenced by the segmentation method.

### Hemodynamics in shear-manipulated RCCA

**Fig. 6:**
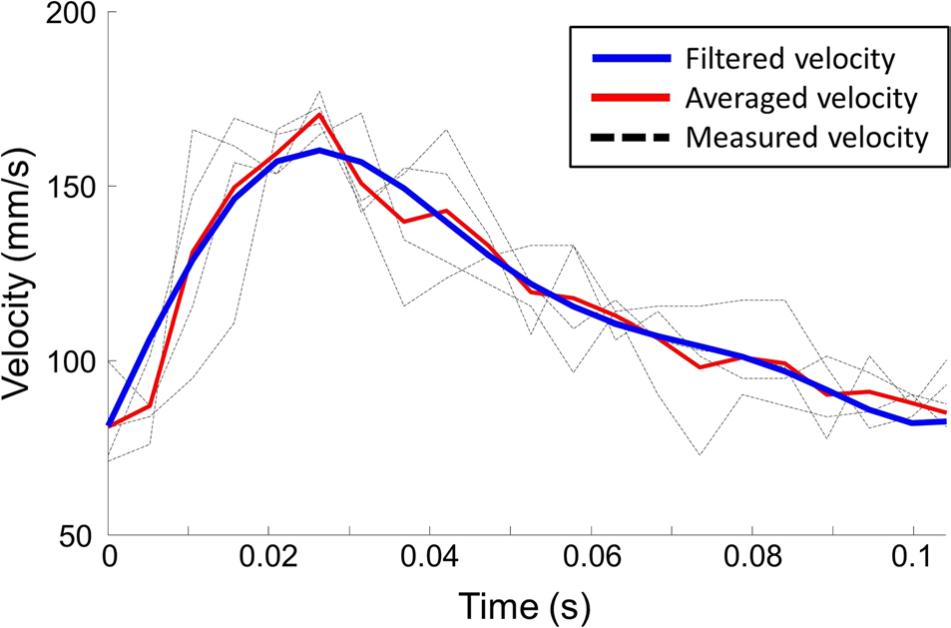
Time-dependent velocity profile at proximal RCCA. *Black dash lines* indicate wave forms measured by Doppler Ultrasound; *Red line* represents the averaged velocity calculated over 4 cardiac cycles while *blue lines* indicates the filtered wave form used as inlet boundary condition

**Fig. 7:**
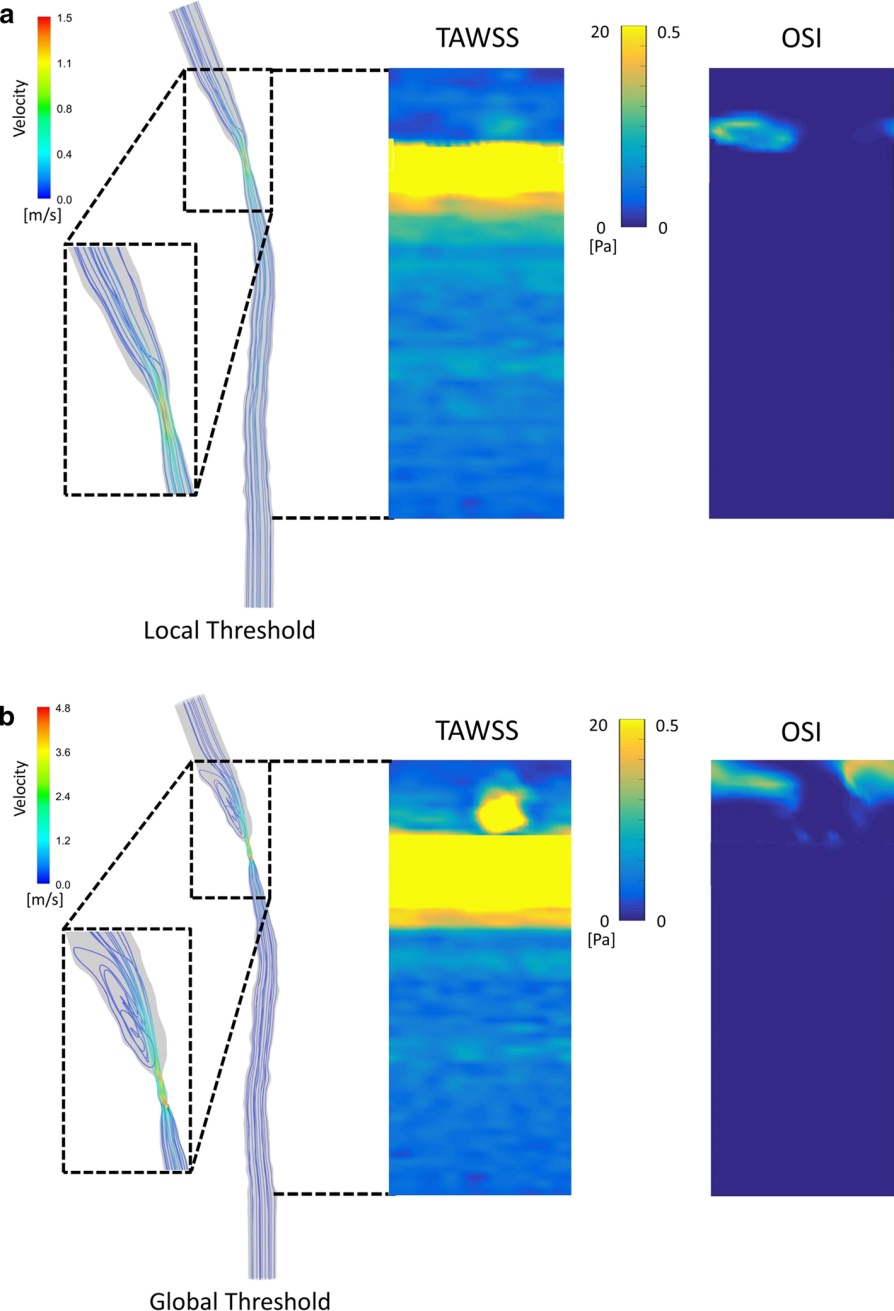
**a** Hemodynamic analysis of shear-manipulated RCCA using vessel geometry created from the local threshold method. Streamline (*left panel*) representing velocity field along the RCCA; TAWSS (*middle panel*) and OSI distribution (*right panel*) along the RCCA. **b** Hemodynamic analysis of shear-manipulated RCCA using vessel geometry created from the global threshold method. Streamline (*left panel*) representing velocity field along the RCCA; TAWSS (*middle panel*) and OSI distribution (*right panel*) along the RCCA. The range of the colormap is between 0 and 20 Pa

Figure 6 shows the velocity field of the blood along the RCCA at the time-averaged velocity in the diastolic phase. Average flow rate and WSS at the inlet of RCCA were summarized in Table 2. Figure 7a (left panel) illustrated the streamlines in the vessel geometry reconstructed from the local threshold method. Upstream of the cast region, parallel streamlines with relatively low velocity was observed, while the flow velocity increased within the cast region. Due to the narrowing of the tapering cast, a slightly asymmetric jet was formed downstream of the cast. Flow reversal downstream of the cast was observed for a short period during peak systole. TAWSS (Fig. 7a, middle panel) was low at the upstream side of the RCCA, with an average value of 6.0 ± 1.8 Pa. Increased TAWSS was seen proximal to the cast region. A maximum TAWSS of approximately 180 Pa was observed inside the cast region where the lumen narrowed. Due to the off-axis jet, asymmetric TAWSS was observed immediately downstream of the cast. Since we did not observe flow reversal upstream of the cast, OSI (Fig. 7a, right panel) was zero, and only the small region downstream of the cast that was exposed to low TAWSS showed elevated OSI levels.

**Table 2:** Average flow rate and wall shear stress at inlet of RCCA

Mouse number	Mean flow rate (mm^3^/s)	Wall shear stress (Pa)
1	25.8 ± 0.2	5.0 ± 0.6
2	15.5 ± 0.1	2.5 ± 0.5
3	N/A	N/A
4	13.7 ± 0.2	1.8 ± 0.2
5	9.4 ± 0.2	1.6 ± 0.2
6	20.2 ± 0.2	3.3 ± 0.2
7	39.0 ± 0.4	6.4 ± 0.1
8	14.8 ± 0.2	2.2 ± 0.1
Average	19.8 ± 9.9	3.3 ± 1.8

In the upstream region, little differences between the local and global threshold method were observed, although stripes of low WSS bands were observed, due to the uneven lumen surface created from the global threshold method (Fig. 7b, middle panel). The global threshold method mainly underestimated the cast dimension (Fig. 5), thus leading to a stronger jet downstream of the cast. This gave rise to flow reversals, even at the time-averaged velocity during the diastolic phase. Furthermore, the jet was asymmetrical, leading to regions with relatively high TAWSS downstream of the cast (Fig. 7b, middle panel). The downstream regions exposed to elevated OSI (Fig. 7b, right panel) were much larger when compared to the results based on the local threshold method.

## Discussion

To monitor changes of WSS over time, knowledge about the vessel geometry at various time points during disease progression is required. The 3D vessel geometry can be acquired by the micro-CT modality by using a contrast agent that provides good contrast enhancement in blood. In our present work, we demonstrated that eXIA 160 provides clear contrast enhancement in the RCCA, thereby facilitating vessel segmentation. Although the amount of the contrast agent administrated was adjusted according to body weight, the contrast intensity in the lumen region varied among animals. We also observed differences in soft tissue contrast intensity among all animals, which was probably caused by the individual differences among the animals. Furthermore, contrast intensity within the cast region also differed among animals. Therefore, it is necessary to analyze contrast enhancement of each animal individually. Moreover, we established a local threshold-based segmentation protocol to reconstruct 3D vessel geometry for calculating WSS. This protocol is especially relevant for WSS analysis in a single mouse over time. Finally, the fact that we observed asymmetric WSS distribution downstream of the cast region suggests that it is preferable to analyze WSS data for each mouse separately.

Global thresholding is a simple segmentation method in vascular image processing for separating blood vessels from the surrounding soft tissue [10, 30, 31]. However, determining vessel geometry using the global threshold method could introduce errors, since the signal variation along the RCCA might then be overlooked. Two of our findings demonstrate that it is indeed better to calculate the threshold value specific to its location along the RCCA. First of all, as the vessel narrowed within the cast region, we observed decreasing contrast intensity in the lumen. This was likely due to the decreasing blood volume within the cast region, thus reducing the amount of contrast agent present in the blood [35]. Consequently, segmentation using global threshold method could underestimate a severely narrowed vessel due to the growth of an atherosclerotic plaque. Secondly, in all the animals, the signal fluctuated along both the lumen region and the background region. This generated a set of fluctuating threshold values that were specific to their location along the RCCA. While this fluctuation artefact was overlooked by segmentation using global threshold method, local threshold segmentation took it into account, thus generating more reliable vessel geometry. Our result was in line with the findings of Vandeghinste et al. [11] in murine aorta without cast. Since the dimension of the cast geometry is known, it was used to validate the results of the two segmentation methods. The vessel surface generated from the global threshold values significantly underestimated the lumen diameter within the cast region when compared to the vessel geometry reconstructed using the local threshold method. Also, in areas outside the cast region, global threshold had lower values than the local threshold method. A lower threshold means that the background region which has lower intensity values could be defined as the vessel region. As a result, this could potentially overestimate the vessel dimensions outside the cast. Therefore, local threshold-based segmentation is a better approach for reconstructing accurate 3D vessel geometry.

Our findings for overall WSS distribution are in good agreement with those of the others: WSS was low upstream of the cast region, with an average value of approximately 6 Pa. Other studies using the same WSS-manipulated mouse model [14, 36] reported a WSS of approximately 10 Pa upstream of the cast. The value that we observed for peak WSS was approximately two-fold lower than values observed by Mohri et al. [14]. The average WSS value downstream of the cast region was also low. No flow recirculation was found using the vessel surface created from the local threshold method. However, due to the slight curvature of the vessel, we found that the vessel wall downstream of the cast was exposed to an asymmetric WSS pattern. A similar observation was reported by Willett et al. [12]. Mohri et al. noted that the recirculation zone covered only a quarter of the wall circumference downstream [14]. In the study performed by Thim et al. [37], in which a non-tapering cast was placed around the carotid artery of minipig, an asymmetrical flow pattern was also evident downstream of the cast as a result of vessel curvature. Once again, this underlines the necessity—when calculating CFD—of performing animal-specific WSS analysis rather than using mouse-averaged vessels or idealized geometries. This concept has also been suggested by van Doormaal et al. [38].

A final consideration in terms of our preference for local threshold-based segmentation when reconstructing accurate 3D vessel geometry is that we observed different WSS patterns downstream of the cast region when global threshold method was used to obtain vessel geometry. As the cast dimension was significantly underestimated, a jet was formed downstream of the cast which led to an unrealistic high WSS distribution in this area. This result illustrates that WSS is highly sensitive to small changes in vessel geometry and thus to the segmentation protocol used for 3D reconstruction. Accurate WSS computation is not only important for the TAWSS but especially for OSI, therefore requires local threshold segmentation.

One of the limitations of our study is that the accuracy of the two threshold methods was only validated within the cast region. For the region outside the cast, we did consider various ways to evaluate the difference between the two segmentation methods. Two approaches potentially qualify for this: high resolution ultrasound and vascular corrosion casting. However, both techniques do not have sufficient resolution to differentiate true dimensions from errors. The difference between the two segmentation methods is small (about 20–30 µm). This small difference can have substantial effects on the WSS computations. However, the spatial resolution of ultrasound is 30 µm, larger than the differences between the two segmentation methods. The use of vascular corrosion casting is another way to obtain vessel dimension. However vessels acquired from this method are known to shrink (16–20%) resulting in an underestimated vessel size [11]. The resolution and errors of the two mentioned alternatives are larger than the differences between the two segmentation methods and therefore cannot be used for validation and drawing robust conclusions. In our study, we only investigated a simple threshold-based segmentation method to reconstruct 3D vessel geometry. The method showed reliable results. We didn’t explore the possibility of using a commercial software package or other more advanced segmentation methods. Whether these methods would give rise to more accurate vessel geometry needs further investigation. Another limitation pertains to the computational methods that were applied to determine the OSI. Recent work has indicated the importance of the buffering capacity of the carotid bifurcation on the computed OSI level, and they concluded that for accurate OSI computations fluid–structure interaction (FSI) simulations were required [39]. Whether FSI simulations would also influence the OSI values immediately upstream of the cast needs to be investigated.

## Conclusion

eXIA 160 provided good blood-pool contrast using a WSS-manipulated atherosclerotic mouse model when performing longitudinal studies. We established a robust segmentation protocol in which local threshold values were applied to generate 3D vessel geometry. The approach eliminated artefact and thus reconstructed reliable vessel geometry for WSS calculation. Finally, we showed that it is crucial to use mouse-specific geometry data when computing WSS.

## References

[1] Lusis AJ (2000). Insight review articles. Nature. Nat Publ Gr.

[2] Ross R (1999). Atherosclerosis–an inflammatory disease. N Engl J Med.

[3] VanderLaan PA, Reardon CA, Getz GS (2004). Site specificity of atherosclerosis: site-selective responses to atherosclerotic modulators. Arterioscler Thromb Vasc Biol.

[4] Slager CJ, Wentzel JJ, Gijsen FJH, Schuurbiers JCH, van der Wal AC, van der Steen AFW (2005). The role of shear stress in the generation of rupture-prone vulnerable plaques. Nat Clin Pract Cardiovasc Med.

[5] Malek AM, Alper SL, Izumo S (1999). Hemodynamic shear stress and its role in atherosclerosis. JAMA.

[6] Slager CJ, Wentzel JJ, Gijsen FJH, Thury A, van der Wal AC, Schaar JA (2005). The role of shear stress in the destabilization of vulnerable plaques and related therapeutic implications. Nat Clin Pract Cardiovasc Med..

[7] Pedrigi RM, Poulsen CB, Mehta VV, Ramsing Holm N, Pareek N, Post AL (2015). Inducing persistent flow disturbances accelerates atherogenesis and promhin cap fotes tibroatheroma development in D374Y-PCSK9 hypercholesterolemic minipigs. Circulation.

[8] Cheng C, Tempel D, van Haperen R, van der Baan A, Grosveld F, Daemen MJAP (2006). Atherosclerotic lesion size and vulnerability are determined by patterns of fluid shear stress. Circulation.

[9] Cheng C, van Haperen R, de Waard M, van Damme LC, Tempel D, Hanemaaijer L (2005). Shear stress affects the intracellular distribution of eNOS: direct demonstration by a novel in vivo technique. Blood.

[10] Suo J, Ferrara DE, Sorescu D, Guldberg RE, Taylor WR, Giddens DP (2007). Hemodynamic shear stresses in mouse aortas: implications for atherogenesis. Arterioscler Thromb Vasc Biol.

[11] Vandeghinste B, Trachet B, Renard M, Casteleyn C, Staelens S, Loeys B (2010). Replacing vascular corrosion casting by in vivo micro-CT imaging for building 3D cardiovascular models in mice. Mol Imaging Biol..

[12] Willett NJ, Long RC, Maiellaro-Rafferty K, Sutliff RL, Shafer R, Oshinski JN (2010). An in vivo murine model of low-magnitude oscillatory wall shear stress to address the molecular mechanisms of mechanotransduction–brief report. Arterioscler Thromb Vasc Biol.

[13] Hoi Y, Zhou Y-Q, Zhang X, Henkelman RM, Steinman DA (2011). Correlation between local hemodynamics and lesion distribution in a novel aortic regurgitation murine model of atherosclerosis. Ann Biomed Eng.

[14] Mohri Z, Rowland EM, Clarke LA, De Luca A, Peiffer V, Krams R (2014). Elevated uptake of plasma macromolecules by regions of arterial wall predisposed to plaque instability in a mouse model. PLoS ONE.

[15] Nebuloni L, Kuhn GA, Müller R (2013). A comparative analysis of water-soluble and blood-pool contrast agents for in vivo vascular imaging with micro-CT. Acad Radiol.

[16] Hallouard F, Anton N, Choquet P, Constantinesco A, Vandamme T (2010). Iodinated blood pool contrast media for preclinical X-ray imaging applications–a review. Biomaterials.

[17] Willekens I, Lahoutte T, Buls N, Vanhove C, Deklerck R, Bossuyt A (2009). Time-course of contrast enhancement in spleen and liver with Exia 160, Fenestra LC, and VC. Mol Imaging Biol.

[18] De Wilde D, Trachet B, Van der Donckt C, Vandeghinste B, Descamps B, Vanhove C (2015). Vulnerable plaque detection and quantification with gold particle-enhanced computed tomography in atherosclerotic mouse models. Mol Imaging.

[19] Rothe JH, Rudolph I, Rohwer N, Kupitz D, Gregor-Mamoudou B, Derlin T (2015). Time course of contrast enhancement by micro-CT with dedicated contrast agents in normal mice and mice with hepatocellular carcinoma: comparison of one iodinated and two nanoparticle-based agents. Acad Radiol..

[20] Boll H, Figueiredo G, Fiebig T, Nittka S, Doyon F, Kerl HU (2013). Comparison of fenestra LC, ExiTron nano 6000, and ExiTron nano 12000 for micro-CT imaging of liver and spleen in mice. Acad Radiol Elsevier.

[21] Boll H, Nittka S, Doyon F, Neumaier M, Marx A, Kramer M (2011). Micro-CT based experimental liver imaging using a nanoparticulate contrast agent: a longitudinal study in mice. PLoS ONE.

[22] Mannheim JG, Schlichthärle T, Kuebler L, Quintanilla-Martinez L, Kohlhofer U, Kneilling M, Pichler BJ. Comparison of small animal CT contrast agents. Contrast Media Mol Imaging. 2016;11:272-84. doi:10.1002/cmmi.1689/full. 10.1002/cmmi.1689 26991457

[23] Detombe SA, Dunmore-Buyze J, Drangova M. Time-course characterization of an aqueous colloidal polydisperse contrast agent in mice using micro-computed tomography. Proceedings of SPIE 7965, medical imaging 2011: biomedical applications in molecular, structural, and functional imaging; 2011. p. 79650M. http://proceedings.spiedigitallibrary.org/proceeding.aspx?articleid=1349955.

[24] Willekens I, Buls N, De Maeseneer M, Lahoutte T, de Mey J (2013). Use of eXIA 160 XL for contrast studies in micro-computed tomography: experimental observations. Mol Imaging..

[25] Lim E, Modi K, Christensen A, Meganck J, Oldfield S, Zhang N (2011). Monitoring tumor metastases and osteolytic lesions with bioluminescence and micro CT imaging. J Vis Exp..

[26] Winkel LCJ, Groen HC, van Thiel BS, Müller C, van der Steen AFW, Wentzel JJ (2014). Folate receptor–targeted single-photon emission computed tomography/computed tomography to detect activated macrophages in atherosclerosis: can it distinguish vulnerable from stable atherosclerotic plaques?. Mol Imaging..

[27] Schaap M, Metz CT, van Walsum T, van der Giessen AG, Weustink AC, Mollet NR (2009). Standardized evaluation methodology and reference database for evaluating coronary artery centerline extraction algorithms. Med Image Anal.

[28] Hameeteman K, Zuluaga MA, Freiman M, Joskowicz L, Cuisenaire O, Valencia LF (2011). Evaluation framework for carotid bifurcation lumen segmentation and stenosis grading. Med Image Anal.

[29] Peiffer V. Study of the relation between blood flow and the age-dependent localisation of atherosclerosis. PhD Thesis. Imperial College London; 2012.

[30] Zagorchev L, Oses P, Zhuang ZW, Moodie K, Mulligan-Kehoe MJ, Simons M (2010). Micro computed tomography for vascular exploration. J Angiogenes Res..

[31] Wirjadi O. Survey of 3d image segmentation methods. Technical Report 123. Fraunhofer ITWM, Kaiserslautern. 2007. https://www.researchgate.net/profile/Syamsiah_Mashohor/publication/220638042_Survey_on_liver_CT_image_segmentation_methods/links/573d11a208aea45ee841a64b.pdf.

[32] Gijsen FJH, Allanic E, van de Vosse FN, Janssen JD (1999). The influence of the non-Newtonian properties of blood on the flow in large arteries: unsteady flow in a 90° curved tube. J Biomech.

[33] Ku DN, Giddens DP, Zarins CK, Glagov S (1985). Pulsatile flow and atherosclerosis in the human carotid bifurcation. Positive correlation between plaque location and low oscillating shear stress. Arteriosclerosis..

[34] Lacolley P, Challande P, Boumaza S, Cohuet G, Laurent S, Boutouyrie P (2001). Mechanical properties and structure of carotid arteries in mice lacking desmin. Cardiovasc Res.

[35] Park E-A, Lee W, Park SJ, Kim YK, Hwang HY (2016). Influence of coronary artery diameter on intracoronary transluminal attenuation gradient during CT angiography.

[36] Kuhlmann MT, Cuhlmann S, Hoppe I, Krams R, Evans PC, Strijkers GJ (2012). Implantation of a carotid cuff for triggering shear-stress induced atherosclerosis in mice. J Vis Exp..

[37] Thim T, Hagensen MK, Hørlyck A, Kim WY, Niemann AK, Thrysøe SA (2012). Wall shear stress and local plaque development in stenosed carotid arteries of hypercholesterolemic minipigs. J Cardiovasc Dis Res..

[38] Van Doormaal M, Zhou Y-Q, Zhang X, Steinman DA, Mark Henkelman R (2014). Inputs for subject-specific computational fluid dynamics simulation of blood flow in the mouse aorta. J Biomech Eng.

[39] De Wilde D, Trachet B, De Meyer G, Segers P (2016). The influence of anesthesia and fluid–structure interaction on simulated shear stress patterns in the carotid bifurcation of mice. J Biomech.

